# Ultrasensitive and ultrastretchable metal crack strain sensor based on helical polydimethylsiloxane

**DOI:** 10.3762/bjnano.15.25

**Published:** 2024-03-01

**Authors:** Shangbi Chen, Dewen Liu, Weiwei Chen, Huajiang Chen, Jiawei Li, Jinfang Wang

**Affiliations:** 1 Shanghai Xin Yue Lian Hui Electronic Technology Co. Ltd, Shanghai, P.R. China; 2 Inertial Technology Division, Shanghai Aerospace Control Technology Institute, Shanghai, P.R. China; 3 Department of Nursing, Shanghai General Hospital, Shanghai Jiao Tong University School of Nursing, Shanghai, P.R. China

**Keywords:** crack sensors, helical structure, polydimethylsiloxane (PDMS), ultrahigh sensitivity, ultrahigh stretchability

## Abstract

The majority of crack sensors do not offer simultaneously both a significant stretchability and an ultrahigh sensitivity. In this study, we present a straightforward and cost-effective approach to fabricate metal crack sensors that exhibit exceptional performance in terms of ultrahigh sensitivity and ultrahigh stretchability. This is achieved by incorporating a helical structure into the substrate through a modeling process and, subsequently, depositing a thin film of gold onto the polydimethylsiloxane substrate via sputter deposition. The metal thin film is then pre-stretched to generate microcracks. The sensor demonstrates a remarkable stretchability of 300%, an exceptional sensitivity with a maximum gauge factor reaching 10^7^, a rapid response time of 158 ms, minimal hysteresis, and outstanding durability. These impressive attributes are attributed to the deliberate design of geometric structures and careful selection of connection types for the sensing materials, thereby presenting a novel approach to fabricating stretchable and highly sensitive crack-strain sensors. This work offers a universal platform for constructing strain sensors with both high sensitivity and stretchability, showing a far-reaching significance and influence for developing next-generation practically applicable soft electronics.

## Introduction

In recent years, there has been significant advancement in the field of stretchable and soft electronic devices due to the increasing demand for their applications in various domains [1–2]. These applications include the detection of human motion [3–5], monitoring human health [6–8], medical treatment [9–10], soft robotics [11–12], and human–computer interaction [13–15]. Numerous flexible strain sensors employing various mechanisms such as piezoresistivity [16–17], capacitance [18–19], and piezoelectricity have been developed to fulfill the demands of these applications [20]. Among various factors considered, the parameters of sensitivity and stretchability hold significant importance in determining the suitability of a strain sensor for practical applications.

In recent years, scholars have acknowledged and addressed the aforementioned challenge by focusing on the structural design of sensing materials in order to enhance both sensitivity and sensing range [21–36]. For instance, Lee et al. successfully developed a strain sensor by utilizing microcracks in a metal nanoparticle thin film deposited on a microstructured polydimethylsiloxane (PDMS) substrate [21]. The sensor exhibits exceptional strain sensitivity, allowing for stretching of up to 20% strain. Liu et al. have successfully developed a strain sensor that exhibits high-performance characteristics [22]. A fish-scale-like microstructure grants the strain sensor exceptional stretchability, a wide sensing range (reaching up to 82% strain), and remarkable sensitivity (with a gauge factor (GF) ranging from 16.2 to 150). In a similar vein, Cai et al. developed strain sensors utilizing a weaving architecture that integrated two-dimensional Ti_3_C_2_T*_x_* MXene nanostacks [23]. The sensor exhibited a high GF of 772.6 when subjected to a strain range of 40–70%, owing to the presence of cracks induced in the MXene layer and carbon nanotubes (CNTs) acting as bridges. In a separate study, Xin et al. reported the fabrication of highly sensitive and stretchable strain sensors with an impressive GF exceeding 42000 at a strain level of 150% [24]. These sensors were created by utilizing precisely controlled cracks in CNT films, which were formed through laser engraving of a CNT paper. In their study, Lee et al. have developed a strain sensor that operated by separating overlapping CNTs embedded within a silicone elastomer [25]. The resulting sliding and disconnection of these CNTs contribute to the exceptional performance of the strain sensor, which demonstrates ultrahigh sensitivity with a GF of 42300 at a strain range of 125–145%. Similarly, Kim et al. propose an approach incorporating a superaligned carbon nanotube sheet between a sensory metal film and an elastomer substrate, resulting in excellent and well-balanced strain sensing performance [26]. This characteristic imparts significant stretchability (ε = 100%) to the Pt crack sensors while simultaneously maintaining an ultrahigh sensitivity (GF ≈ 12274). Sun et al. developed a novel strain sensor by combining double-layer micropatterned Au and SWCNTs to achieve a strain sensor with a GF as high as 3.4 × 10^6^ under 100% strain [27]. He et al. successfully fabricated a sandwich structure consisting of a sensing layer composed of CNTs and MXene on a flexible thermoplastic polyurethane substrate [34]. The strain sensor exhibits exceptional sensing range (390%) and sensitivity (GF = 2159.5). These findings highlight the importance of rational design of geometric structures and control of connection types of sensing materials as effective strategies to achieve such desirable characteristics. However, limited research has explored the potential of helical structures for achieving extensive stretchability in ultrasensitive sensors.

This paper introduces a stretchable resistive sensor that exhibits both high sensitivity and a wide range of strain through the combined integration of a cracked thin metal and a 3D helical substrate. The fabrication process involves depositing a Au thin film onto a PDMS substrate with helical structure, followed by pre-stretching to induce microcracks in the Au thin film. The resistance of the sensor is altered when strain is applied because of the separation of overlapping scales and the generation of cracks in the gold thin film. The strain sensor is fabricated through a straightforward preparation method, resulting in an exceptionally high gauge factor of 10^7^, a broad strain range of 300%, a rapid response time of 158 ms, minimal hysteresis, and outstanding durability. (The GF serves as a means to assess the sensitivity of stretchable strain sensors; it is defined as the ratio of the relative change in resistance to the applied mechanical strain, expressed as GF = [(*R* − *R*_0_)/*R*_0_]/ε. Here, *R* represents the resistance observed during stretching, *R*_0_ denotes the initial resistance, and ε signifies the magnitude of the mechanical strain applied.) This versatile sensor not only accurately detects small physiological signals such as human joint movement and identifies variations in ambient temperature, but it can also monitor diverse large deformation movements in real time, such as those involved in the mechanical control of security alert systems. To the best of our knowledge, this study represents the first examination of a metal film with cracks applied onto a helical elastomer substrate, with a specific focus on evaluating its electromechanical capabilities.

## Results and Discussion

### Morphology and structure

**Figure 1:**
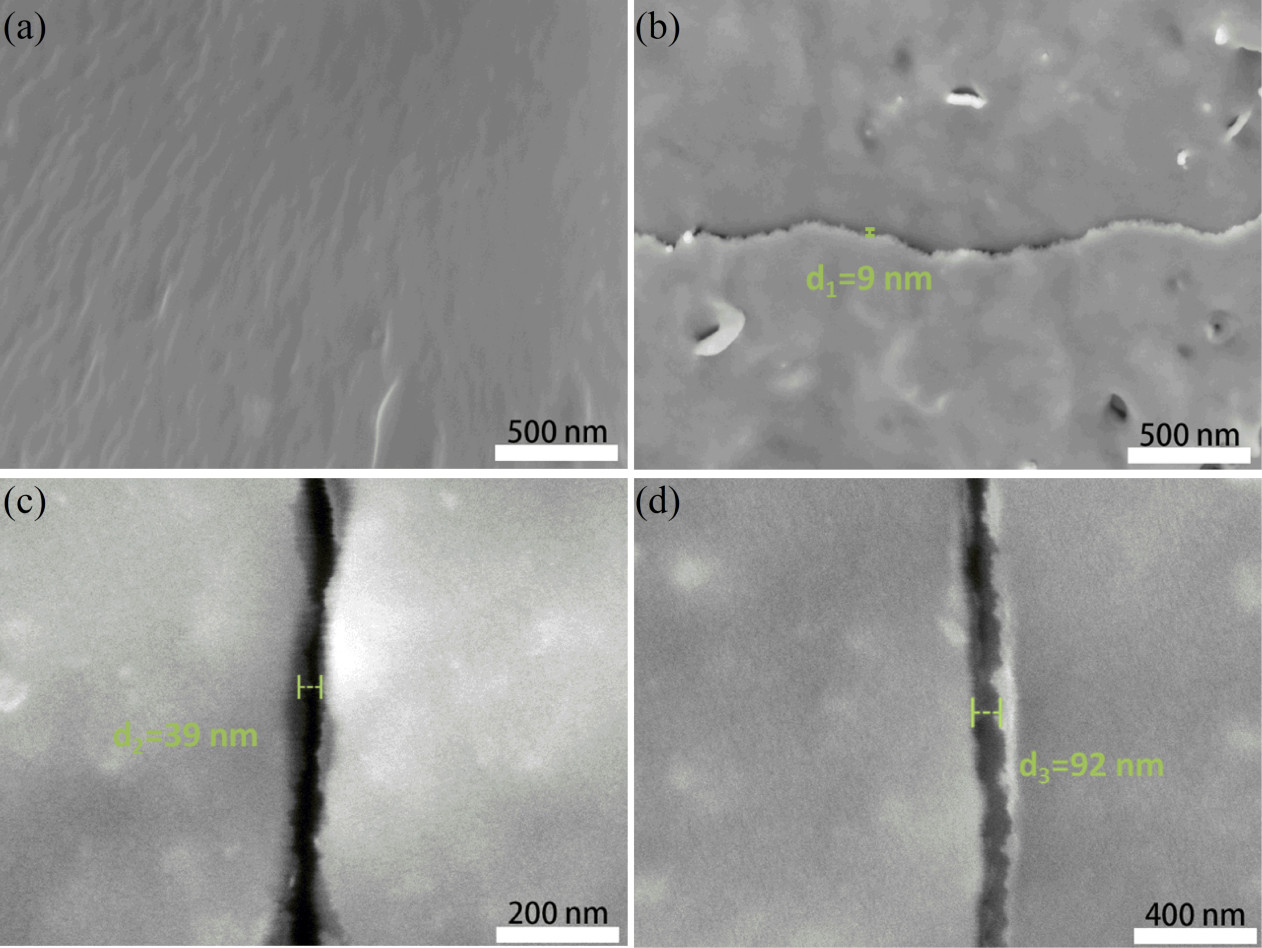
SEM images of tunnel cracks with different width formed under strain. (a) SEM images of the metal crack strain sensor with no cracks. (b) Cracks form. (c) Cracks grow. (d) Cracks widen.

Initially, the microcracks in the metal film overlap after straightening the helix and pre-stretching at a strain of 10%. Subsequently, upon release, cracks form randomly, as depicted in Figure 1a. During the tensile loading process, as shown in Figure 1b, two neighboring gold strips lose contact, resulting in the gradual generation of gaps accompanied by a few isolated microcracks. With further stretching, an increase is observed in Figure 1c. Concurrently, the microcracks propagate into channel cracks that traverse the entire width of the sample, as illustrated in Figure 1d. The crack structure evolution of the helix metal film under tensile strain ranging from 0 to 300% was observed using an optical microscope, as depicted in Figure S1 (Supporting Information File 1). These cracks serve to separate the conductive medium of the metal layer from the PDMS substrate, thereby influencing the width of the conductive tunnels and the mode of conduction. Widening of the cracks and a decrease of conductive paths occur when tension is applied. Consequently, the resistance of the helical sensor gradually increases. In order to comprehend this characteristic, an examination of the resistance behavior of the crack structure was conducted using a basic electrical circuit model, as shown in Figure S2 (Supporting Information File 1). Equation 1 is employed to analyze the resistance behavior of the crack structure:


[1] $$R=\frac{R_{1}R_{2}}{R_{1}+2R_{2}}.$$

The resistances of the Au film islands and the Au bridges between two adjacent film islands are denoted as *R*_1_ and *R*_2_, respectively. As the strain on the sensor increases, the cracks in the Au film widen, leading to a reduction of current paths between the Au islands. This reduction in the number of effective Au bridges that can electrically connect the ruptured film portions under high strain results in a rapid increase in total resistance. Figure S3 (Supporting Information File 1) illustrates the measurement of different initial resistances for helical samples during different stretching cycles, which can be attributed to the alteration in crack number and crack width due to the fatigue of the metal film. Following 0–160 cycles of stretching, the resistance of the sample exhibits a significant increase from 6.23 Ω (with an error range of approximately 5%) to 116.52 Ω. However, during the subsequent stretching period of 160–220 cycles, the resistance remains relatively stable, ranging from 116.52 to 117.49 Ω. Based on these observations, it is inferred that quantity and size of the cracks in the helical samples remain relatively constant.

### Strain response of the sensor

**Figure 2:**
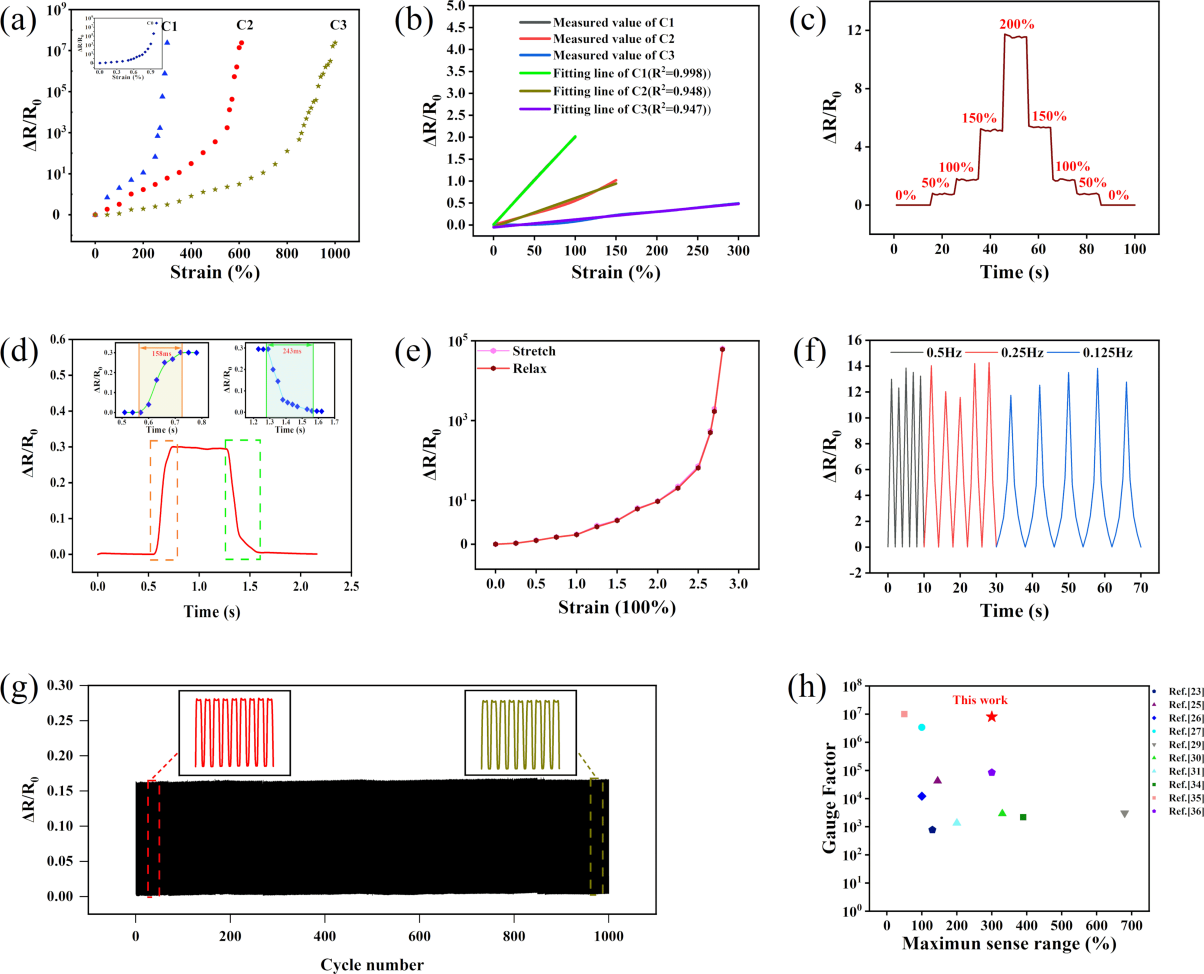
Strain-sensing performance of the metal crack strain sensor. (a) Relative resistance change as a function of the applied strain with helix indices of 1, 2, and 3. Insets: relative resistance change as a function of the applied strain in the flat gold film. (b) The linearity between the relative resistance changes and strain. (c) Change in resistance for a step strain from 0 to 200%. (d) Response time of the strain sensor. Insets: close-ups of the selected areas. (e) Hysteresis curve of the helical strain sensor at 280% strain. (f) Frequency tests at an applied strain of 0 to 200%. (g) Durability test for 1000 stretch–release cycles under 150% strain. (h) Comparison of maximum working range and GF of the helical sensor with recent publications.

As depicted in Figure 2a, the resistance of the metal film on the helical surface of the PDMS substrate undergoes a relative change (Δ*R*/*R*_0_) upon stretching, where Δ*R* and *R*_0_ represent the initial resistance and the transient resistance, respectively. The resistance changes exhibit a gradual increase initially, followed by a steep increase once a certain strain threshold is exceeded, with variations observed for different helix indices (*C* is the ratio of the helical sensor diameter *D* to the helical fiber diameter *d*). Notably, the resistance curves display a conspicuous “J-shape” pattern with two discernible stages, as demonstrated in Figure S4 (Supporting Information File 1). The resistance error range of the samples was observed during stretching in Figure S5 (Supporting Information File 1), indicating the reproducibility of metal crack formation under stretching. The sensors with helix indices C1, C2, and C3 exhibited distinct turning points at 250%, 550%, and 850% strain, respectively. The dominance of strain in the sensing mechanism can be attributed to the tunneling effect [37]. As the Au film is stretched, it gradually separates, resulting in an increased distance between adjacent cracks. During this stage, the resistance is primarily influenced by the coupling of tunneling resistance with the physical distance between channel cracks. Therefore, the change in resistance, corresponding to the average width of the cracks and the strain, can be described by the following formula:


[2] $$In\frac{R}{R_{0}}=-In^{1+\varepsilon }+Xd\varepsilon .$$

When the strain ε is small, we can formulate:


[3]

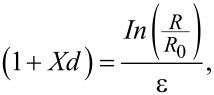



where *X* is the tunneling barrier height-dependent function. Figure 2b shows the good linearity between measured resistance and strain; the curves fit quite well to the analytical solution. When the helical index is increased, the turning point of the sensor exhibits an expansion, consequently leading to an enlargement of the overall linear range. As depicted in Figure 2c, the helical strain sensor underwent testing under dynamic strain conditions. The sensor was exposed to strain from 0 to 200%, followed by a return to 0% strain, with each increment being 50% and a dwell time of 10 s. The relative resistance exhibited a gradual increase as the strain was increased from 0 to 200%; it subsequently reverted to its initial level upon release of the strain from 200 to 0%. The helical sensor demonstrated remarkable stability and favorable recoverability. Figure 2d illustrates the response time of the helical sensor when subjected to a quasi-transient step strain of 10%. Notably, the response time was approximately 158 ms, while the relaxation time was approximately 243 ms, indicating a strong response to loading and unloading attributed to the viscoelastic properties of PDMS. Moreover, the sensor exhibited exceptionally rapid response behavior, further affirming its reliability. The hysteresis of the helical sensor was assessed at a strain of 280% and a stretching speed of 10% per minute in a complete cycle, as depicted in Figure 2e. The stretch and release curves, which are nearly identical, show remarkably low hysteresis and minimal structural damage during the cycling experiment. This observation further substantiates the favorable responsive recoverability of the helical sensor. The influence of strain rate dependence is of considerable significance in the realm of strain sensors. In Figure 2f, the output signals of the helical sensor were recorded at a strain of 200% and various frequencies of 0.125 Hz, 0.25 Hz, and 0.5 Hz. The results demonstrate a consistent change in relative resistance within the tested frequency range, indicating the helical sensor’s sensitivity and stability across a wide range of frequencies. Furthermore, the helical sensor exhibits dynamic durability, as it maintains a steady electrical response and mechanical integrity during long-term stretch and release cycles. The samples were subjected to 1000 cycles of stretching and releasing with a strain of 150%, and the resulting changes in relative resistance were recorded, as depicted in Figure 2g. There is a decrease of approximately 5% in the output signal; this gradual attenuation phenomenon has been previously observed in crack-based strain sensors [37]. It is evident that the helical strain sensor exhibits exceptional stability and recoverability, thereby demonstrating excellent reproducibility and durability in practical applications. In Figure 2h, the helical sensor demonstrates exceptional strain-sensing capabilities over a wide operational range (300%) and with remarkable sensitivity, surpassing the performance of previously reported strain sensors based on structural designs. (The sensitivity of stretchable strain sensors is commonly expressed via the GF, which is defined as the proportion between the relative alteration in resistance and the applied strain. Here it is up to 10^7^.) These findings suggest that the incorporation of helical structures in strain sensors could greatly enhance the trade-offs between sensing indicators.

### Application

Leveraging the unique characteristics of the helical structure, the sensor could be effectively employed to differentiate between varying degrees of finger bending. Based on prior studies, the expression for the bending inductance is as follows [38]:


[4] $$H_{b}=k\times \frac{\pi \mu_{0}N^{2}D^{2}}{2\left[1+\sqrt{1-{\left(\frac{D}{2R}\right)}^{2}}\right]L_{0}}.$$

**Figure 3:**
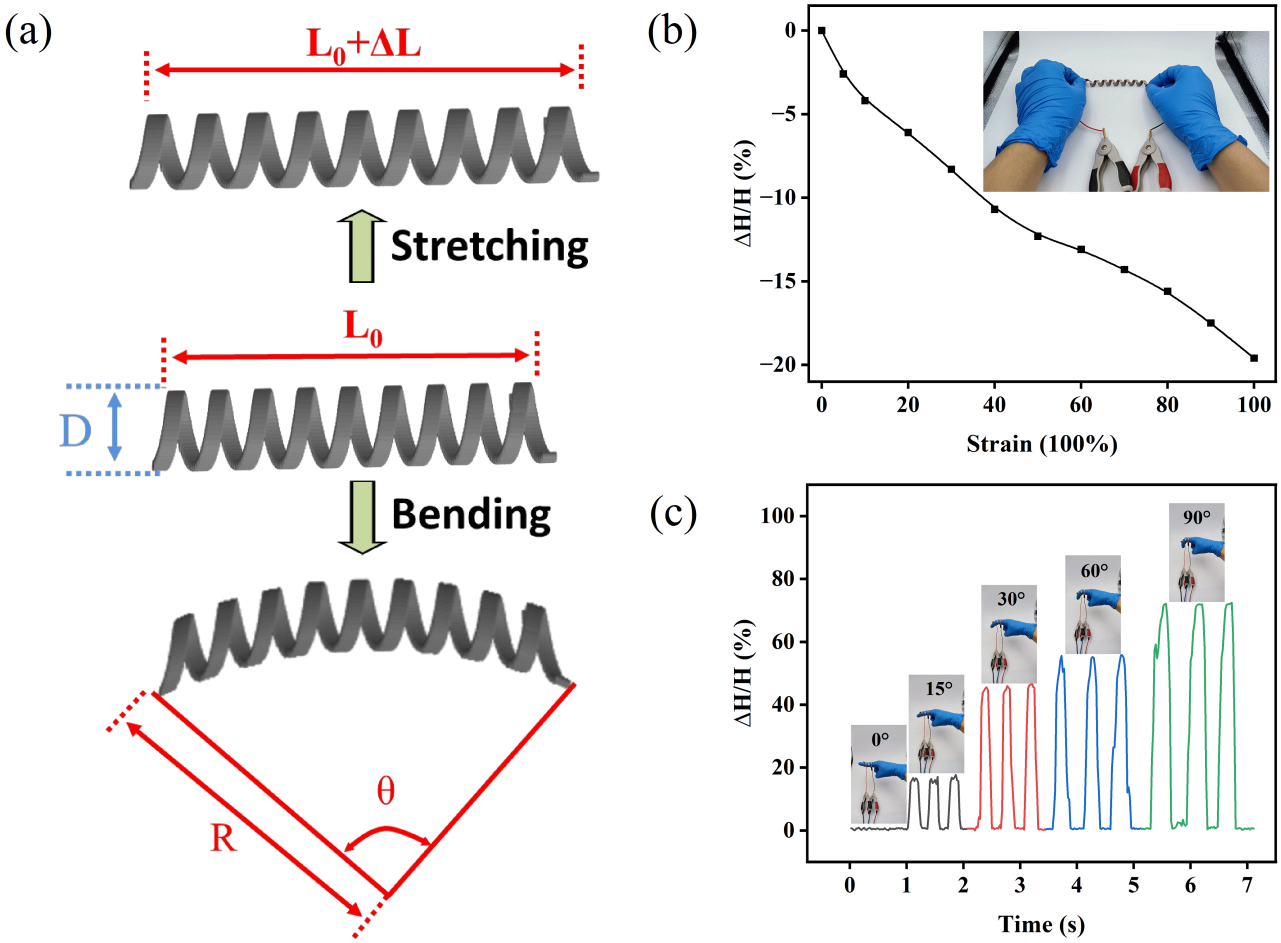
(a) Schematic illustration of the helical inductive sensor. (b) The change in inductance of the helical sensor as a function of the strain. (c) The response of the helical inductive sensor to different extents of finger bending.

*D* and *L*_0_ denote the initial coil diameter and the length of the helical sensor, respectively. μ_0_ is the permeability of vacuum (4π × 10^−7^ N·A^−2^), and *k* is the aspect ratio correction factor, which relies on the ratio between coil diameter *D* and length *L*_0_, as shown in Figure 3a. Figure 3b depicts the alteration in inductance as a function of the strain. The helical index of this sensor is 1, while its length is 2 cm. The inductance of the sensor diminishes as the degree of stretching intensifies, aligning with Equation 4. Furthermore, Figure 3c visually demonstrates noticeable fluctuations in inductance corresponding to the level of finger flexion. Consequently, the sensor showcases rapid and accurate responsiveness in quantifying the extent of flexion.

**Figure 4:**
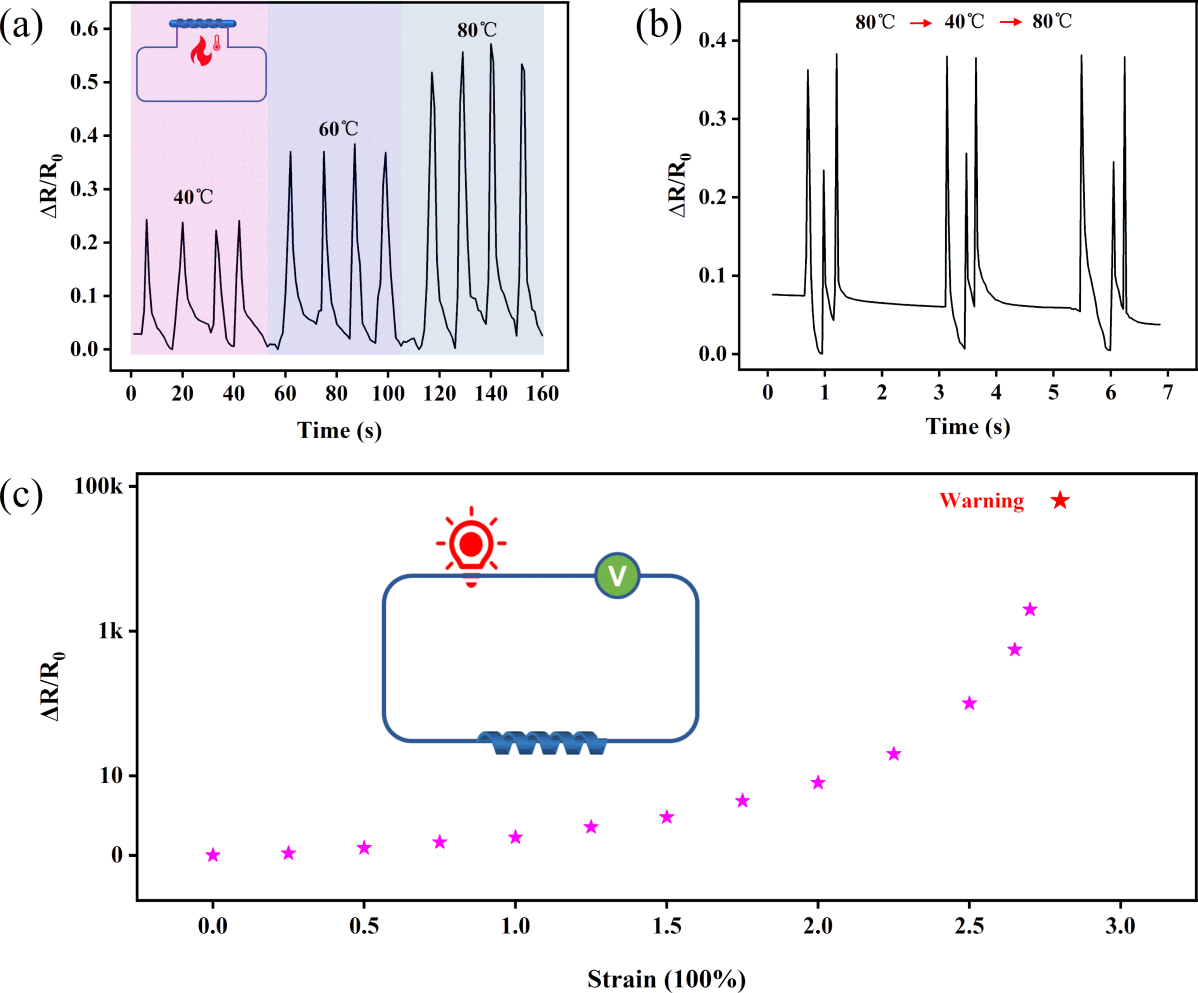
(a) Δ*R*/*R*_0_ of the helical sensor as function of the temperature. (b) The relative resistance change varies with the external temperature. (c) The different brightness of an LED as alarm signal. Inset: the LED in series with the strain sensor.

Figure 4a illustrates the relationship between relative resistance change and temperature. A mandrel wrapped by the helical sensor undergoes expansion under external heating, resulting in alterations in helical diameter and resistance output. The findings indicate that the sensor possesses enhanced capability in detecting variations in ambient temperature and effectively monitoring them. Furthermore, the sensor exhibits prompt and accurate perception in response to fluctuations in environmental temperature, as depicted in Figure 4b. To showcase the efficacy of the sensor, we devised an overstrain alarm utilizing a strain sensor with initially low resistance. Upon encountering excessive strain, the alarm is triggered. This implementation is illustrated in Figure 4c, where the strain sensor is connected in a simple series circuit with a 3 V power supply and a light-emitting diode (LED). When a tensile strain is applied, the light emitted by the LED is modulated by the strain sensor. The LED rapidly switched off when the sensor surpassed the critical strain threshold of the target. The switch-off of the LED could serve as an alarm signal, and the threshold strain required to trigger this alarm could be adjusted by manipulating the helix indices of the helical sensor.

## Conclusion

In summary, this study presents a straightforward method for producing a remarkably flexible resistive strain sensor by utilizing a Au thin film on a helical structure. The fabrication process involves depositing a gold thin film onto a PDMS substrate with helical structures, obtained from mold processing, followed by pre-stretching to induce microcracks in the Au thin film. Curvature and torsion of the helix significantly contribute to the redistribution of surface strains in the elastomer during stretching and play a vital role in the functionality of the strain sensor. Based on its helical microstructure, the sensor demonstrates an ultrahigh gauge factor of 10^7^, along with a wide strain range of 300%, a rapid response time of 158 ms, minimal hysteresis, and remarkable durability. This versatile sensor not only accurately detects subtle physiological signals, such as human joint movement. It is also effective in determining changes of the environmental temperature and enables mechanical control mechanisms for security alert systems. This article presents an innovative approach through the development of a strain sensor based on cracks with high stretchability, aiming to address the limitations of sensing range in soft electronic devices. Furthermore, this research provides a versatile framework for constructing strain sensors that exhibit both high sensitivity and stretchability, thereby demonstrating significant implications and potential for the advancement of practical soft electronics in the next generation.

## Experimental

### Materials

Polydimethylsiloxane (PDMS, Sylgard 184) was provided by Dow Corning Co. Ltd. (Michigan, USA). The screws and hollow tubes were acquired from the taobao shopping platform. The medical rubber gloves and injection syringes were procured from a local hospital (Shanghai, China). The titanium (Ti) target (99.99%) utilized for the adhesion layer was purchased from Deyang Ona New Materials Co., Ltd. The gold (Au) target (99.999%) employed for ion beam sputtering was obtained from Fuzhou Yingfei Xun Photoelectric Tech Co., Ltd, China; it possessed a density of 19.3 g·cm^−3^ and a conductivity of 4.52 × 10^7^ S·m^−1^. Silver conductive adhesive, which was procured from Shenzhen Ausbond Co., LTD. (Guangdong, China), was employed to affix copper wires as electrodes on both sides of the helical polydimethylsiloxane.

### Fabrication of the metal crack strain sensor

**Figure 5:**
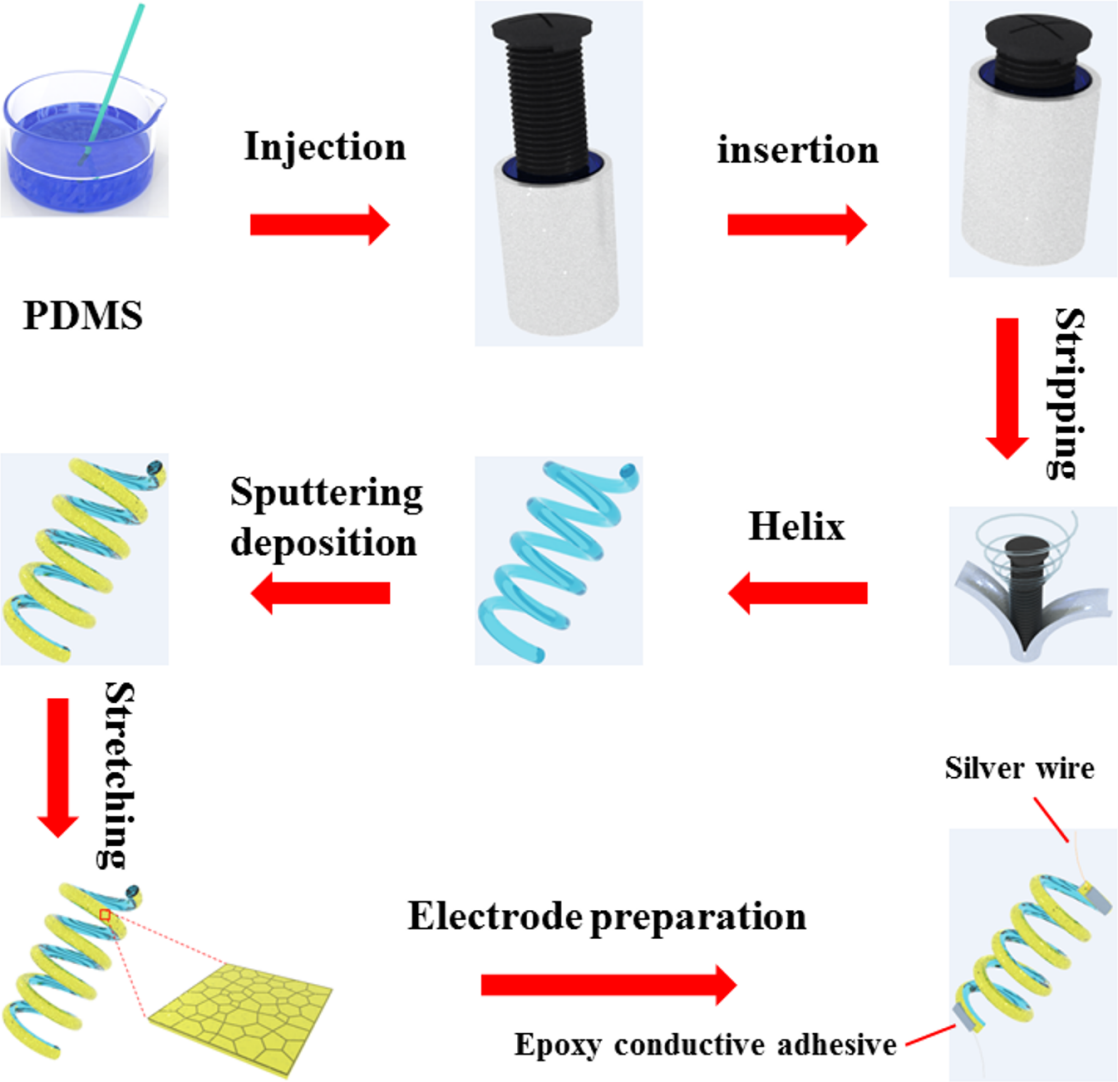
Schematic illustration of the fabrication process of the metal crack strain sensor based on helical polydimethylsiloxane.

The helical structure of PDMS was achieved by utilizing a screw as a mold, as depicted in Figure 5. To ensure the absence of bubbles within the grooves of the screw, a precisely fitting hollow tube was employed for filling the PDMS. The elimination of air bubbles was accomplished through the application of a vacuum pump. Subsequently, the screw was inserted into the PDMS-filled straw by rotating it. The screw’s outer diameter and the straw’s inner diameter exhibit a favorable compatibility, leading to a gradual extrusion of PDMS upon rotational movement of the screw. This process ensures the absence of any voids or air bubbles. Then, the PDMS was cured at a temperature of 100 °C for 1 h. Following the curing process, the tube was detached, and the helically structured PDMS was successfully retrieved by peeling it off from the screw. In order to eliminate the contaminants from the surface of the helically structured PDMS, a 10 min ultrasound treatment in absolute alcohol was employed, followed by drying in a sterile oven. Prior to the Au deposition, the outer surface of the helically structured PDMS was initially cleansed through a 5 min Ar plasma sputtering and, subsequently, coated with a 10 nm thick Ti adhesion layer. After that, ion beam sputtering was utilized to deposit a 50 nm thick Au thin film onto the outer surface of the helically shaped PDMS. Then, a controlled pre-stretch was exerted on the surface of the Au film at a rate of 10% strain per second, resulting in the initiation and propagation of cracks. Following the release of the pre-stretch, the Au thin films were affixed to silver wires using silver paste at both ends. Ultimately, the surface of the helix was coated with PDMS. Parts of the experimental procedure are shown in Figure S6 (Supporting Information File 1).

### Characterization

The surface morphology of the samples prepared in this study was examined through the utilization of scanning electron microscopy (SEM) (TESCAN MIRA3, Czech Republic) and an optical microscope (TD-4KH, Shenzhen Sanqtid Optical Instrument Co., China). Tensile properties were assessed using a computer-controlled high-precision single-axis tensile tester (ZQ-990B) in conjunction with a digital source meter (Keithley DMM6500, USA) to record resistance values during the stretching process.

## Supporting Information

File 1 Additional experimental data.

## Data Availability

All data that supports the findings of this study is available in the published article and/or the supporting information to this article.
