# Supplementary material for: Ultrasensitive and ultrastretchable metal crack strain sensor based on helical polydimethylsiloxane
Source: Beilstein J Nanotechnol. 2024 Mar 1;15:270–8. doi: 10.3762/bjnano.15.25 (PMC10910384; doi:10.3762/bjnano.15.25)
Supplement: File 1 — Additional experimental data. [file Beilstein_J_Nanotechnol-15-270-s001.pdf]

BEILSTEIN JOURNAL OF NANOTECHNOLOGY

Supporting Information

for

Ultrasensitive and ultrastretchable metal crack strain sensor based on helical polydimethylsiloxane

Shangbi Chen, Dewen Liu, Weiwei Chen, Huajiang Chen, Jiawei Li and Jinfang Wang

Beilstein J. Nanotechnol. **2024,** *15,* 270–278. doi:10.3762/bjnano.15.25

Additional experimental data

License and Terms: This is a supporting information file under the terms of the Creative Commons Attribution License (https://creativecommons.org/licenses/by/4.0). Please note that the reuse, redistribution and reproduction in particular requires that the author(s) and source are credited and that individual graphics may be subject to special legal provisions.
The license is subject to the *Beilstein Journal of Nanotechnology* terms and conditions: (https://www.beilstein-journals.org/bjnano/terms)

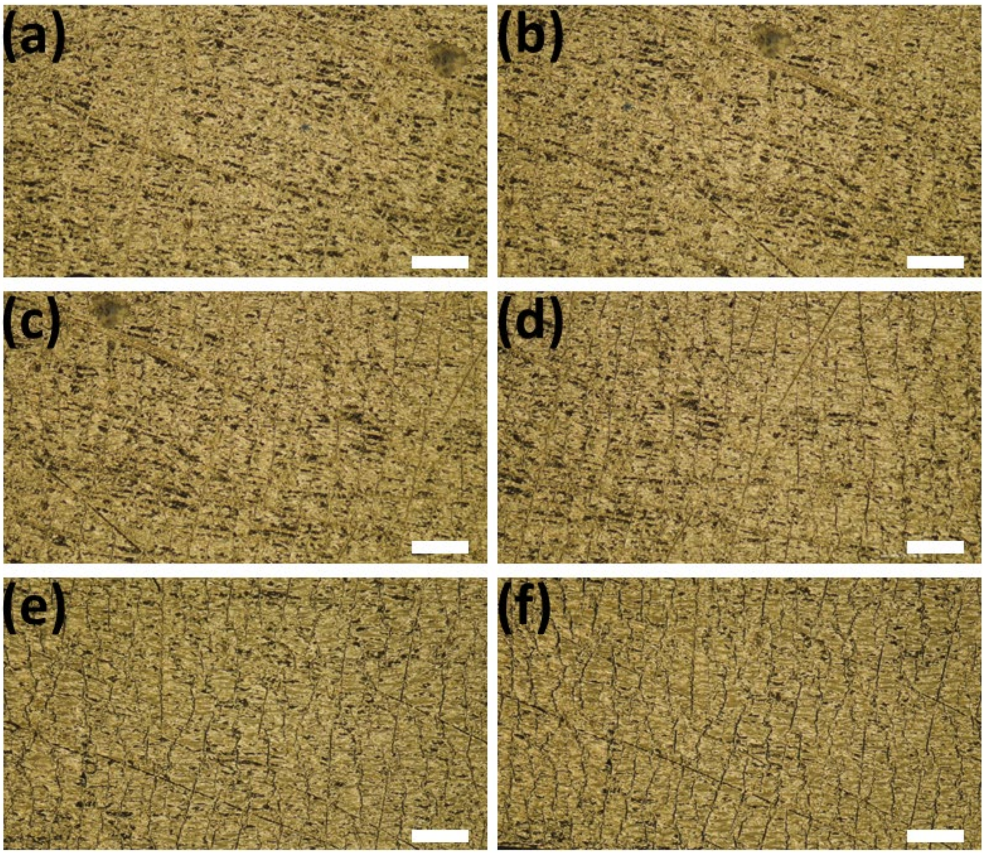

Figure S1: Schematic illustration of the fabrication process of the metal crack strain sensor based on helical polydimethylsiloxane. Scale bars are 200 μm.

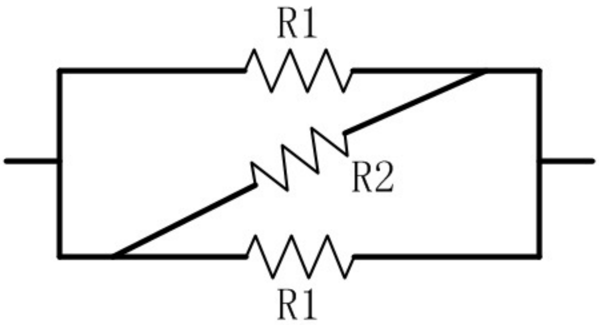

Figure S2: Electrical circuit model of the helical sensor.

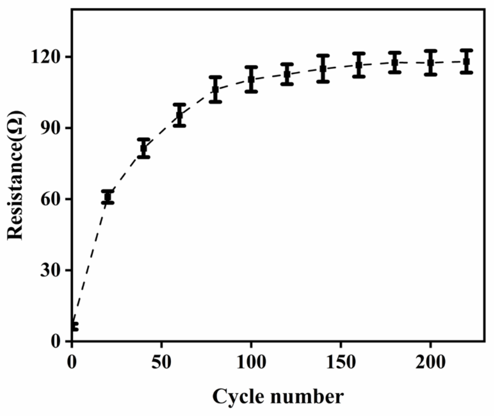

Figure S3: Variation of resistance of helical samples with forming channel cracks under repeated stretching with 10% pre-stretch.

Helical design

Helical microstructures exhibit “J-shaped” stress–strain behavior [1-3], and the geometry is described by the contour length (L), pitch (p), radius (R), width (w) and thickness (t), as shown in Figure S3. The conditions of $t/w \ll 1$ and $w/L \ll 1$ were fulfilled in this paper. Thus, the helical PDMS is much stiffer to bending deflections along the width than deflections normal to its thickness. A series of analytic expressions were developed by Pham and co-workers [1]. A term for the bending force can be obtained:

$$F_{bend} = \left(\frac{B}{2}\right)\frac{8\pi^2 p}{l^3}\left[\frac{\sqrt{\left(1-\left(p_0/l\right)^2\right)}}{\sqrt{\left(1-\left(p/l\right)^2\right)}}-1\right] \tag{S1}$$

where p_0 and *B* are the initial pitch of the helix and the bending stiffness, respectively. l is the contour length of a single turn. The twisting force can be defined as follows:

$$F_{twist} = \left(\frac{C}{2}\right)\frac{8\pi^2 p}{l^3} \tag{S2}$$

where *C* is the twisting stiffness. We combine Equation S1 and Equation S2,

and the total force is found to be:

$$F_{total} = \frac{E\pi^2 wt^3 p}{3l^3}\left[\frac{\sqrt{\left(1-\left(p_0/l\right)^2\right)}}{\sqrt{\left(1-\left(p/l\right)^2\right)}} + \frac{1-\nu}{1+\nu}\right]$$

where $B = Ewt^3/12$ and $C = 2B/(1+\nu)$. E and ν are the elastic modulus and Poisson's ratio of the material, respectively.

From this equation, the non-linear mechanics response force increases as the pitch increases upon extension until the pitch increases to its maximum extension $(p = l)$.

Helices can effectively suppress strain concentration because of the small physical coupling to the substrate. Under the applied strain, the helix meritoriously absorbs the strain, which leads to highly elastic mechanical behavior [4].

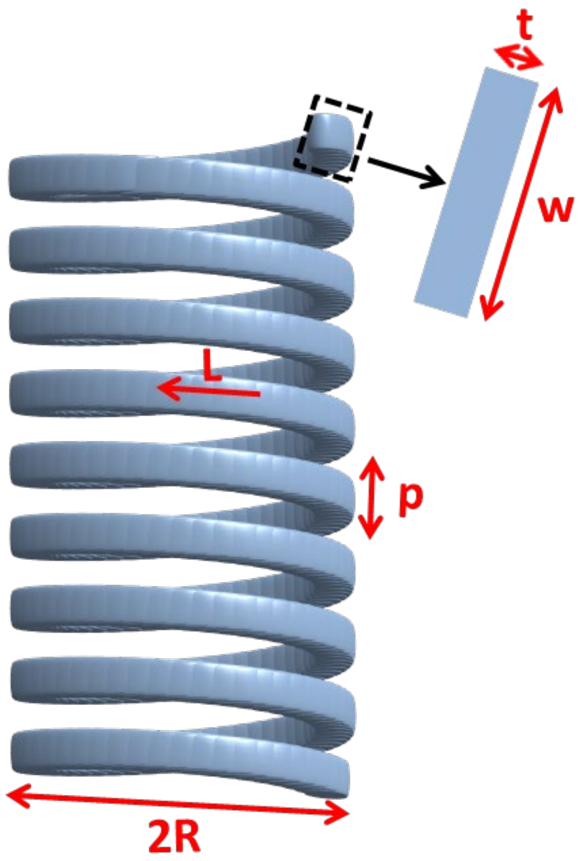

Figure S4: Geometrical illustration of the helical microstructure.

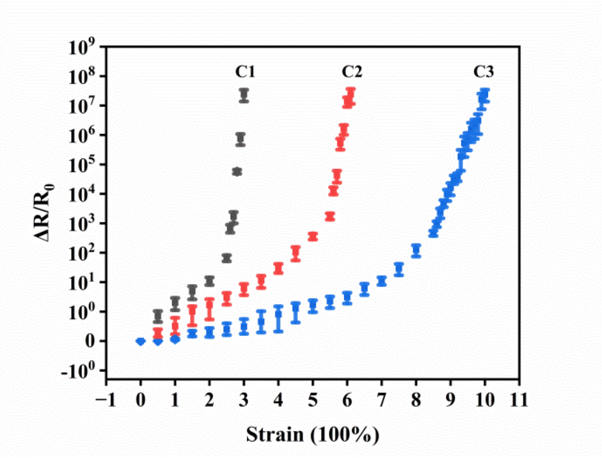

Figure S5: The resistance error range of samples upon stretching.

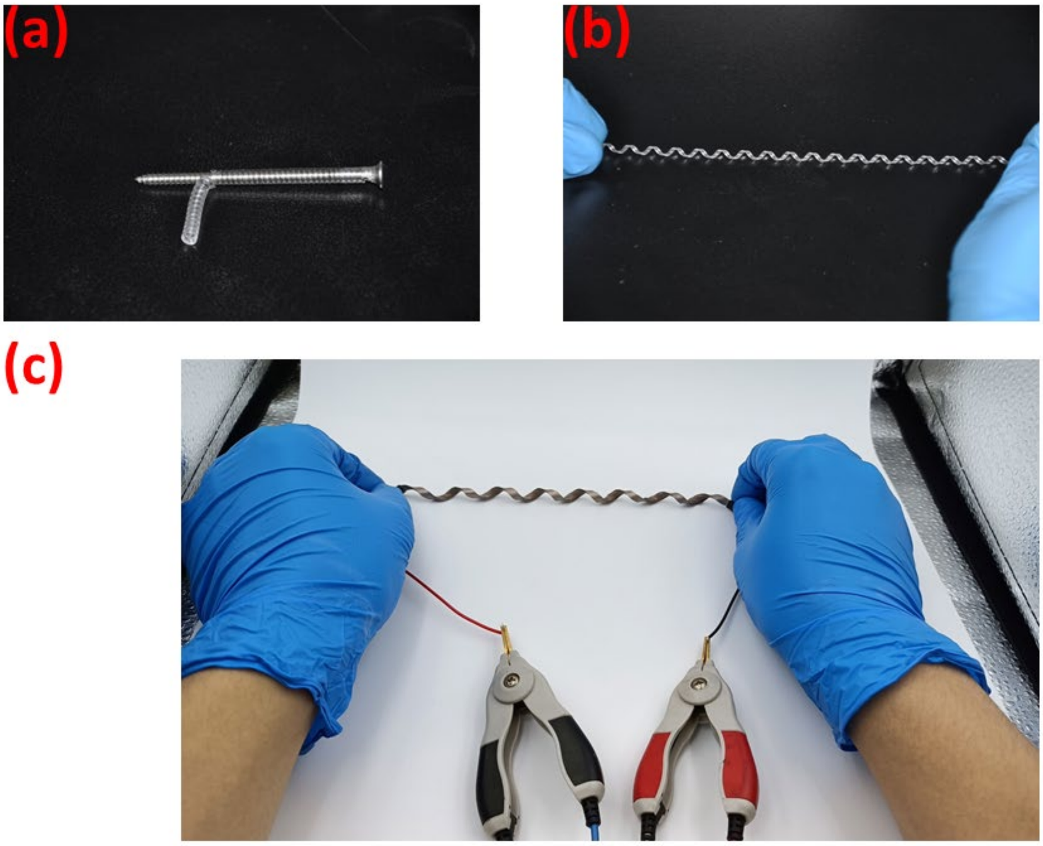

Figure S6: Images of parts of experimental procedure.

References

1. Pham, J. T.; Lawrence, J.; Grason, G. M.; Emrick, T.; Crosby, A. J. *Phys Chem Chem Phys*, **2014**, *16*, 10261-6. doi: 10.1039/c3cp55502j
2. Pham, J. T.; Lawrence, J.; Lee, D. Y.; Grason, G. M.; Emrick, T.; Crosby, A. *J. Adv Mater*, **2013**, *25*, 6703-8. doi: 10.1002/adma.201302817
3. Ma, Y.; Feng, X.; Rogers, J. A.; Huang, Y.; Zhang, Y. *Lab Chip*, **2017**, *17*, 1689-1704. doi: 10.1039/c7lc00289k
4. Jang, K. I.; Li, K.; Chung, H. U.; Xu, S.; Jung, H. N.; Yang, Y.; Kwak, J. W.; Jung, H. H.; Song, J.; Yang, C.; Wang, A.; Liu, Z.; Lee, J. Y.; Kim, B. H.; Kim, J. H.; Lee, J.; Yu, Y.; Kim, B. J.; Jang, H.; Yu, K. J.; Kim, J.; Lee, J. W.; Jeong, J. W.; Song, Y. M.; Huang, Y.; Zhang, Y.; Rogers, J. A. *Nat Commun*, **2017**, *8*, 15894. doi: 10.1038/ncomms15894
